# Supplementary material for: Comparative Analysis of PRNP Gene Indel Polymorphism and Expression among Zhongdian Yellow Cattle, Zhongdian Yak, and Their Hybrids
Source: Animals (Basel). 2023 Nov 23;13(23):3627. doi: 10.3390/ani13233627 (PMC10705791; doi:10.3390/ani13233627)
Supplement: Supplementary file 1 [file animals-13-03627-s001.zip › Suplementary Figure.pdf]

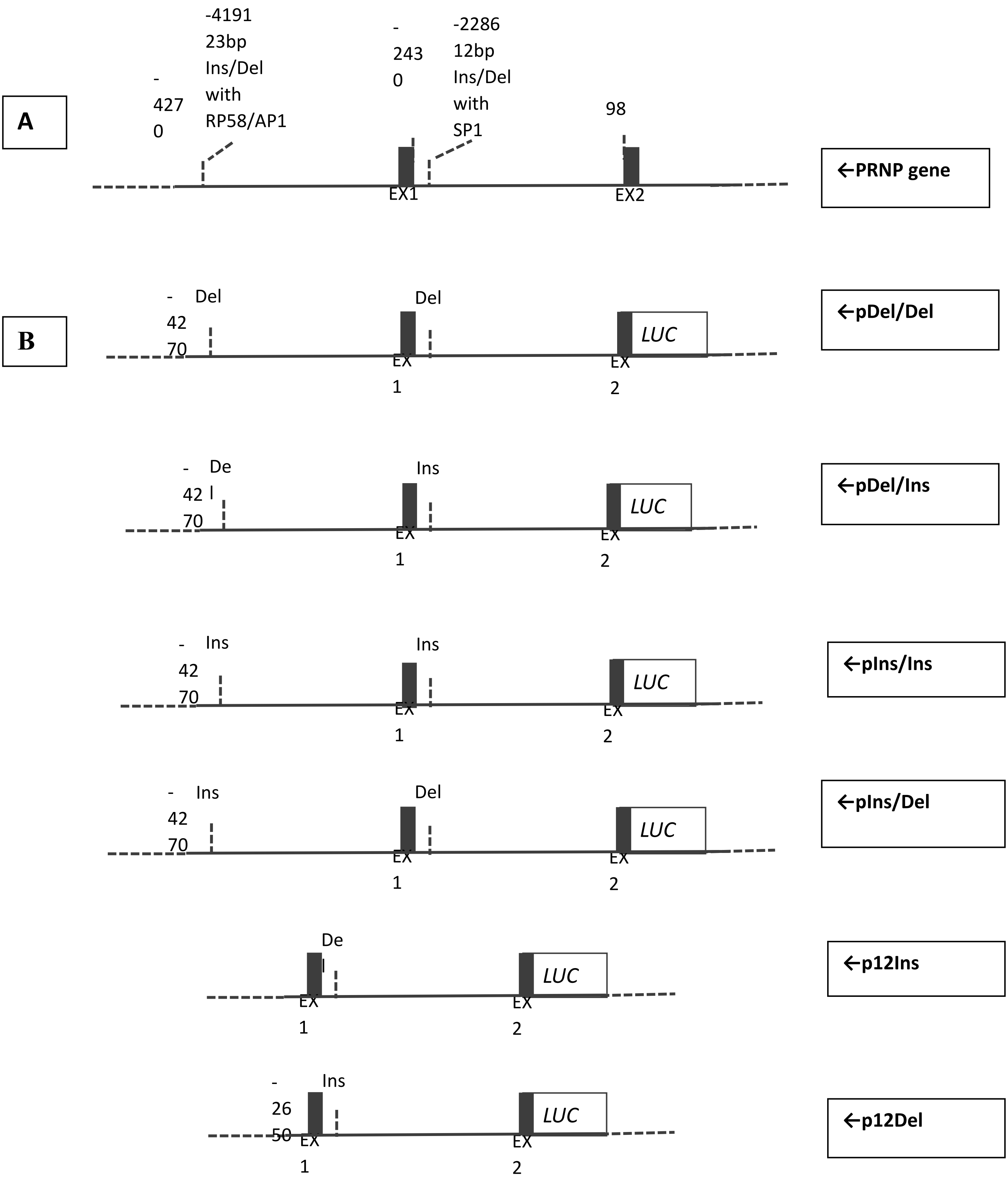
A
-4270
-4191 23bp Ins/Del with RP58/AP1
-2430
-2286 12bp Ins/Del with SP1
98
EX1
EX2
←PRNP gene
B
-4270
Del
Del
EX 1
EX 2
LUC
←pDel/Del
-4270
Del
Ins
EX 1
EX 2
LUC
←pDel/Ins
-4270
Ins
Ins
EX 1
EX 2
LUC
←pIns/Ins
-4270
Ins
Del
EX 1
EX 2
LUC
←pIns/Del
Del
EX 1
EX 2
LUC
←p12Ins
-2650
Ins
EX 1
EX 2
LUC
←p12Del

Figure S1. (**A,B**) Schematic representation of the 5-end of the bovine *PRNP* gene and the reporter gene constructs used in this study. (A) the first two untranslated exons of the bovine *PRNP* gene are indicated EX1 & EX2. Three transcription factors whose binding sites are affected by the polymorphisms are indicated. (B) six reporter gene constructs in the pGL3-Basic vector were prepared. In these constructs, various alleles of the bovine *PRNP* promoter drive the expression of firefly luciferase.
