# Supplementary material for: Comparative Analysis of PRNP Gene Indel Polymorphism and Expression among Zhongdian Yellow Cattle, Zhongdian Yak, and Their Hybrids
Source: Animals (Basel). 2023 Nov 23;13(23):3627. doi: 10.3390/ani13233627 (PMC10705791; doi:10.3390/ani13233627)
Supplement: Supplementary file 1 [file animals-13-03627-s001.zip › Suplementary Tables.pdf]

Table S1. The reaction condition of PCR

Loci	Pre-denaturation	Denaturaion	Anealing	Extension	Final Extension	No: of cycles
PRNP-12 indel	94^0C/5min	94^0C/1min	56^0C/45sec	72^0C/1min	72^0C/7min	36
PRNP-23 indel	94^0C/5min	94^0C/1min	58^0C/45sec	72^0C/1min	72^0C/7min	38

Table S2. List of Primers and target genes using in this study

Genes	sequence	Size	AT ^{0}C	Reference
PRNP	F 5'-CTTCTCTCTCGCAGAAGCAG-3'	414/42	54	Nakamistu *et al*, 2006
	R 5'CCCTTGTTCTTCTGAGCTCC3'			
PRNP	F 5'-AAGGCACTTCAATCAGTACAC-3'	130/15	53	Brunelle *et al*, 2008
	R 5'-AAGAGTTGGACAGGCACAATG-3'			
PRNP	F 59-TCCCAGAGACACAAATCCAA-39	153	60	Msalya *et al*, 2011
	R 59-ATCCTCCTCCAGGTTTTGGT-39			
	F 59-TCCAACTTGAGCTGAATCACA-39	153	60	
	R 59-CAGGTTTTGGTCGCTTCTTG-39			
ACTB	F 59-ACCATGTACCCCGGCATC-39	169	60	Msalya *et al,* 2011
	R 59-TTGCTGATCCACATCTGCTG-39			
	F 59-ATCGAGGACAGGATGCAGAA-39	154	60	
	R 59-CACATCTGCTGGAATGTGGA-39			

**Table S3.** The sequences used for EMSA probes.		
Primer	sites	Sequence
Bio23^{+}	AP1	5' Biotin-TCAATCTCAGATGTCTTCCCAACAGCAGCC
Bio23^{-}	-	5' Biotin-GCTATCACGTCAAGCCTCAGACGTCAT
Cold23^{+}	AP1	5'-TCAATCTCAGATGTCTTCCCAACAGCAGCC
Mut23^{+}	-	5'-TCAATCTCAGATGTCTTCGGAACAGCAGCC
Bio12^{+}	SP1	5' Biotin-TGGGCGGGGGCCGCGGCTGGCTG
Bio12^{-}	-	5' Biotin-TCGGAATGTGGGCTGGCTGGTCC
Clod12^{+}	SP1	5'-TGGGCGGGGGCCGCGGCTGGCTG
Mut12+	-	5'-TGGGCGGGTGACACGGCTGGCTG
Notes: Bio=Biotin, Met=Mutant.		

Table S4. Analysis of the Ins/Del fragments that affects transcriptional factor-biding sites.

23bp

5 MATalpha2[T00487]	6 HMG1(Y)[T02368]	7 STAT4 [T01577]
13 NF-1[T00536]	14 NF-1[T00538]	15 TGGCA-binding protein [T00832]
21 HNF-3beta [T02344]	22 HOXA3[T00378]	23 MYB2[T02536]
29 PR B [T00696]	30 PR A[T01661]	31 GR-alpha [T00337]
37 NHP-1[T00621]	38 RP58[T05040]	

0 Myf-3[T00519]	1 MyoD[T00525]	2 MyoD[T01128]	3 INSAF[T00406]	4 NF-X3[T015141]
8 p300[T01427]	9 FACB[T02841]	10 YY1[T04970]	11 e-Ers-1[T00112]	12 MF3[T00507]
16 R2[T00712]	17 LIM1[T04817]	18 C/EBPalpha[T00107]	19 AP-1[T01150]	20 STAT6[T01581]
24 NF1/CTF[T00094]	25 CREMtau[T01309]	26 CREMtau1[T02108]	27 CREMtau2[T02109]	28 MEF1[T00506]
32 COE1[T01112]	33 LyF-1[T00479]	34 STAT5A[T04683]	35 Elk-1[T00250]	36 Pax-6[T00682]

12bp

0 ZF5[T02349]	1 ETF[T00270]	2 E2F-1[T01542]	3 f(alpha)-f(epsilon)[T00287]
4 p53[T00671]	5 Sp1 [T02338]	6 BTEB4[T05053]	7 MYBASI[T05553]

Note:The representative AP-1, RP58 and SP-1 transcriptional factors are indicated with red arrows.
